# Novel cost-effective design for bio-volatilization studies in photosynthetic microalgae exposed to arsenic with emphasis on growth and glutathione modulation

**DOI:** 10.3389/fmicb.2023.1170740

**Published:** 2023-06-19

**Authors:** Atul K. Upadhyay, Shekhar Mallick, Ranjan Singh, Lav Singh, Nitesh Singh, S. K. Mandotra, Arpit Singh, Ravi Prakash Srivastava, Shivaraman Pandey, Gauri Saxena

**Affiliations:** ^1^Department of Environmental Science, School of Earth & Environmental Sciences, Babasaheb Bhimrao Ambedkar University, Lucknow, India; ^2^Plant Ecology and Environmental Science, National Botanical Research Institute, Lucknow, India; ^3^Central Academy for State Forest Services, Burnight, Assam, India; ^4^Forest Training Institute, Kanpur (Ministry of Environment, Forest and Climate change, Govt. of Uttar Pradesh, India; ^5^Department of Plant Pathology, Faculty of Agricultural Sciences, Shree Guru Gobind Singh Tricentenary University, Gurugram, India; ^6^Department of Botany, Panjab University, Chandigarh, India; ^7^Department of Botany, Lucknow University, Lucknow, Uttar Pradesh, India; ^8^Government PG College, Datia, Madhya Pradesh, India

**Keywords:** volatilization, algae, arsenic, biotransformation, *Nannochloropsis* sp., *A. doliolum*, glutathione

## Abstract

A novel laboratory model was designed to study the arsenic (As) biotransformation potential of the microalgae *Chlorella vulgaris* and *Nannochloropsis* sp. and the cyanobacterium *Anabaena doliolum*. The Algae were treated under different concentrations of As(III) to check their growth, toxicity optimization, and volatilization potential. The results revealed that the alga *Nannochloropsis* sp. was better adopted in term of growth rate and biomass than *C. vulgaris* and *A. doliolum.* Algae grown under an As(III) environment can tolerate up to 200 μM As(III) with moderate toxicity impact. Further, the present study revealed the biotransformation capacity of the algae *A. doliolum*, *Nannochloropsis* sp., and *Chlorella vulgaris*. The microalga *Nannochloropsis* sp. volatilized a large maximum amount of As (4,393 ng), followed by *C. vulgaris* (4382.75 ng) and *A. doliolum* (2687.21 ng) after 21 days. The present study showed that As(III) stressed algae-conferred resistance and provided tolerance through high production of glutathione content and As-GSH chemistry inside cells. Thus, the biotransformation potential of algae may contribute to As reduction, biogeochemistry, and detoxification at a large scale.

## 1. Introduction

Rapid industrialization, mining, tailings, and modern agro-farming have led to toxic element pollution in the environment, frequently destructing soil health and having negative consequences on the wellbeing of people and the ecosystem. The toxic elements present in the soil can be improved by the use of plants and the microorganism present in soil through the process of green technology, which is one of the most appealing and popular methods for leveraging natural processes to breakdown, gather, and stabilize pollutants by serving as filters or traps (Raklami et al., 2022; Singh et al., 2022, 2023a). Heavy metal contamination of the biosphere has been increasing as a result of accelerated human activity, and it is becoming a major worldwide concern. The use of biological processes to remove such inorganic contaminants from aquatic environments has gained a lot of favor because of their high efficiency and cost-effectiveness when compared to traditional physicochemical approaches (Ranjbar and Malcata, 2022; Kumar et al., 2023; Singh et al., 2023b). Heavy metals and metalloids inhibit growth and reproduction due to the consequent physiological and metabolic abnormalities, thus lowering crop productivity. Therefore, they must alter their mode of adaptation to endure in such a hostile environment without compromising their ability to grow, develop, reproduce, or survive. The process of adaptation involves an intricate network of signaling cascades that control gene expression at the transcriptional and post-transcriptional levels, altering and adapting the biochemical and physiochemical parameters (Kumar et al., 2020, Pradhan et al., 2022).

Arsenic (As) is a persistent environmental toxin (Yin et al., 2011a,b; Ye et al., 2012) that exists in different oxidation states and varies with environmental conditions, including inorganic As(III and V) in drinking water and organic As in foods (Naujokas et al., 2013). The major route of As contamination in the ecosystem is the use of As-laden groundwater, mining of base metals, food processing, and fertilizers (Chen et al., 2009). The food chain contamination of As leads to cell death in plants, while in humans prolonged exposure may even cause mortality (Meliker et al., 2007). A large-scale epidemiological study in Bangladesh showed that exposure to As from drinking water contributed 21% chronic-disease mortalities (Argos et al., 2010).

The presence of the *ars* (arsenic resistance) gene from prokaryotes to archaea reflects its widespread occurrence, and it confers resistance to As. In eukaryotes, As resistance is achieved by glutathionylation coupled with the complex [As(GS)_3_] extrusion from the cytosol by the transporter present in the cells (Qin et al., 2009). The biotransformation of As through microbes may play a detrimental role in As biogeochemical cycling and As bioremediation from wastewater and contaminated soil. The phenomenon of As volatilization is in chorus with accumulation, including the transformation of As [MAs(V), DMAs(V), TMAsO(V)] to more volatile organic arsenical [TMAs (III)] (Qin et al., 2009; Yin et al., 2011a,b). The toxic side of As(III) causes turgidity loss, membrane disruption, and cell death in plants, which are counterbalanced by different tolerance mechanisms (enzymatic and non-enzymatic antioxidants) present inside the cell. The non-antioxidants, *viz.*, glutathione (GSH), play a significant role in As(III) metabolism and remediation by forming the As(III)-GSH complex in the cytoplasm and exporting by the ABC transporter (Scott et al., 1993; Qin et al., 2009) from the cell.

Algae are ubiquitously present in water and soil contaminated with metals (Ye et al., 2012). They can accumulate a significant amount of metals in its body mass. Past studies have already proven the positive role of the microorganism and plants in bioremediation and controlling levels of heavy metals. The features that make them ideal candidates for the selective removal and concentration of heavy metals include their rapid growth rate, high tolerance to heavy metals and changing environment, luxuriant growth both autotrophically and heterotrophically, large surface area/volume ratios, phytochelatin expression, and potential for genetic manipulation (Lee and Wang, 2001; Pisciotta et al., 2010). In recent years, several species of the green and blue green algae have been utilized to assess metal content in many parts of the world (Yin et al., 2011a,b; Abdel-Raouf et al., 2012).

To date, most studies of microbial communities have focused on bacteria and archaea, with little attention given to eukaryotic microorganisms. Photosynthetic microorganisms such as blue-green algae and green algae for their role in As oxidation, reduction, methylation, biotransformation, and bio-volatilization are still underutilized, and As volatilization through photosynthetic microbes is largely unexplored. In the present study, the algae *Anabaena doliolum, Chlorella vulgaris,* and *Nannochlorposis* sp. were used to investigate their volatilization potential through a laboratory-designed cost-effective model. The volatilization potential of microalgae could play a positive role in As cycling, especially in developing countries, in an ecofriendly manner.

## 2. Materials and methods

### 2.1. Collection and culture of microalgae

The alga *Nannochloropsis* sp. was collected from As-contaminated areas of West Bengal, while the algae *A. doliolum* and *C. vulgaris* were collected from wastewater-contaminated sites of Gomti river, Lucknow, India. The algal samples were isolated and cultured in a 250 mL Erlenmeyer flask in an extensively used BG-11 nutrient media (pH 6.8) (Rippka et al., 1979). The BG-11 nutrient media consisted of macronutrients including NaNO_3_, MgSO_4_.7H_2_O, K_2_HPO_4_, CaCl_2_.2H_2_O, citric acid, ferric ammonium citrate, Na_2_.EDTA, Na_2_CO_3_, and micronutrients, *viz.*, H_3_BO_3_, MnSO_4_.H_2_O, ZnSO_4_.7H_2_O, CuSO_4_.5H_2_O, and (NH4)_6_MoO_4_.4H_2_O. Algae cultures were maintained in controlled laboratory conditions (27 ± 2°C) under 150 μmol photon/m^2^/sPAR (photosynthetically active radiation) with a photoperiod of 14:10 h (light: dark). The algal cultures were shuddered manually 2–3 times daily. For the optimized growth of the algae, pH of the medium was adjusted to 7.5 with the help of a Tris HCl buffer.

### 2.2. Preparation of the test solution

The test solution of As(III) of different concentrations (50–2,000 μM) was prepared by directly dissolving the required amount of the test substance in the medium. Approximately 5–10% of pure algal inoculums were added and kept for 72 h for growth and acclimatization. All the experiments were conducted in triplicate under controlled laboratory conditions.

### 2.3. Growth and toxicity analysis

The growth of the algae was analyzed by measuring optical density (OD) after 24 h of interval with the help of a spectrophotometer at a wavelength of 700 nm. A toxicity test using algae was intended for the optimization of the As toxicity and growth by exposing the organisms to As dissolved in a culture medium. The toxicity analysis of microalgae treated with different concentrations of As(III) was performed by calculating the lethal concentration (LC 50).

### 2.4. Data analysis

#### 2.4.1. Average growth rate

Average growth was calculated using the formula below (Fogg and Thake, 1987):


$$\mu =\ln\;\left(C_{2}\right)-\ln\;\left(C_{1}\right)/t_{2}-t_{1}.$$

where C_1_ and C_2_ are cell density at the final exponential phase (t_2_) and start phase (t_1_) of the test, respectively.

#### 2.4.2. Specific growth rate

Specific growth rate was calculated using the following equation (Wong and Chang, 1991):


$$\mathrm{Specific growth rate}\;\left(k\right)={\mathrm{lnX}}_{1}-\ln\;X_{2}/T_{1}-T_{0}.$$

where X_0_ = Initial absorbance and X_2_ = Absorption at time T_1_.

#### 2.4.3. Percentage inhibition

The percentage inhibition (PI) of the algae was determined by the formula below:


$$\mathrm{PI}=C_{0}-\mathrm{Ct}/C_{0}\times 100.$$

where C_0_ and Ct were cell density in the control algae and treated group (at stationary phase), respectively.

### 2.5. Trapping and detection of volatiles

**Figure 1:**
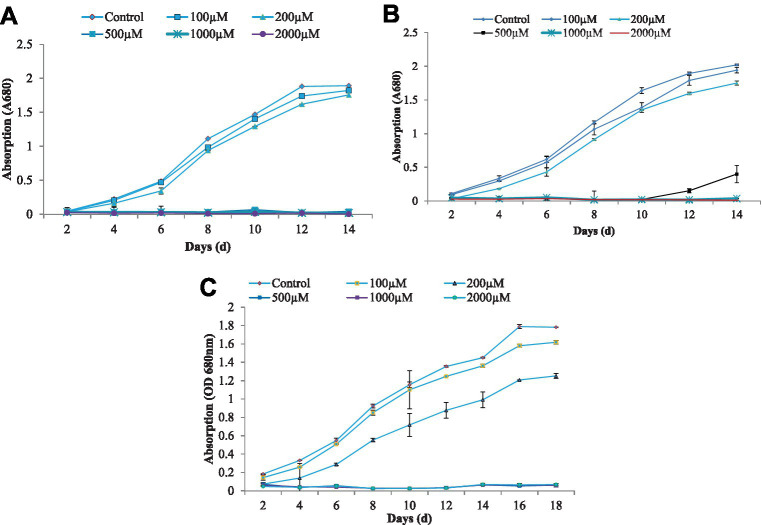
The growth characteristics of algae *C. vulgaris* **(A)**, *Nannochloropsis* sp. **(B)**, and *Anabaena doliolum* **(C)** treated with different concentration of As(III). All values are means ± SD.

The volatile form of arsenic was trapped and determined as reported by Mestrot et al. (2009), with slight modification. This slight modification included altering the size of the silica gel, use of a glass rod instead of a burette of different size and diameter, and use of a volumetric flask with its mouth covered with cork with two holes for the inlet and outlet in order to make the flask airtight. Parafilm was also used to avoid leakage. The model for trapping the volatile As was prepared in the laboratory using an Erlenmeyar flask (1 L), silica tube (3.0 mm diameter), glass rod (length 30 cm diameter 1.2 cm), rubber cork, magnetic shaker, glass rod stand, quartz wool, and air pump. The model was arranged as depicted in Figure 1 under controlled laboratory conditions. The trapping system was prepared was as follows: the silica gel (60–120 mesh size) was saturated with 5% HNO_3_ for 24 h in a 500 mL glass beaker and rinsed with distilled water. After 2–5 times washing, the water was decanted. They were soaked overnight in 10% AgNO_3_ solution (w/v) and then dried in an oven at 80°C. The dried silica gel was further loaded in a glass rod, with one side kept closed by a small quantity of quartz wool. After loading the silica gel, the other end was also packed with wool. The trapper was covered with black cotton cloth and aluminum foil to evade the photodecomposition of AgNO_3_. The mass culture of algae was grown in a 1 L BG 11 medium (Rippka et al., 1979) supplemented with 200 μM As(III). The desired concentration of As(III) was obtained by growth optimization and a toxicity test under different concentrations of arsenic. The flask (5 L) containing the algal culture was made airtight with the help of rubber cork slotted with two hollow glass rods for the inlet and outlet. The inlet was connected with a hushed adjustable air pump (SO- Fine-1 Aqua pump) to supply oxygen for the growth of the algae. The outlet of the designed model was fitted with the one end of the trapping system using a silica tube. The other end was left for air exhaust. The trapping period lasted for different time periods of 7, 14, and 21 days. At the end of the experiment, trapped As was eluted by passing 1% hot boiling HNO_3_ (20 mL) through a trapper and collected in a tube. The total As content was estimated in ICP MS (7500cx, Agilent Technology, United States). The entire procedure since elution was kept under low light and cold conditions.

### 2.6. Estimation of GSH

Glutathione content was estimated by the method of Anderson (1985). A total of 50 mg fresh centrifuged algal sample was homogenized in 3 mL of 5% sulphosalicylic acid under cold condition. It was then centrifuged at 10,000 rpm for 10 min, and 0.5 mL aliquot was mixed with 0.5 mL reaction buffer (0.1 M phosphate buffer, pH 7.0, and 0.5 mM EDTA) and 50 μL 3 mM DTNB [5,5′-dithio-bis-(2-nitrobenzoic acid)]. The absorbance of the reaction mixture was recorded after 10 min at the wavelength of 412 nm.

### 2.7. Estimation of pigment

The pigment content in the microalgae was estimated by the methods of Arnon (1949). A total of 25 mg fresh algal biomass was crushed in chilled 2 mL acetone (80%v/v) with a mortar and pestle. The mixture was centrifuged at 8,000 × g for 10 min. After centrifugation, the supernatant was extracted, and total chlorophyll, chlorophyll a, and chlorophyll b were estimated by taking absorbance at 663 and 645 nm.

## 3. Results

### 3.1. Growth characteristics of the algae

**Figure 2:**
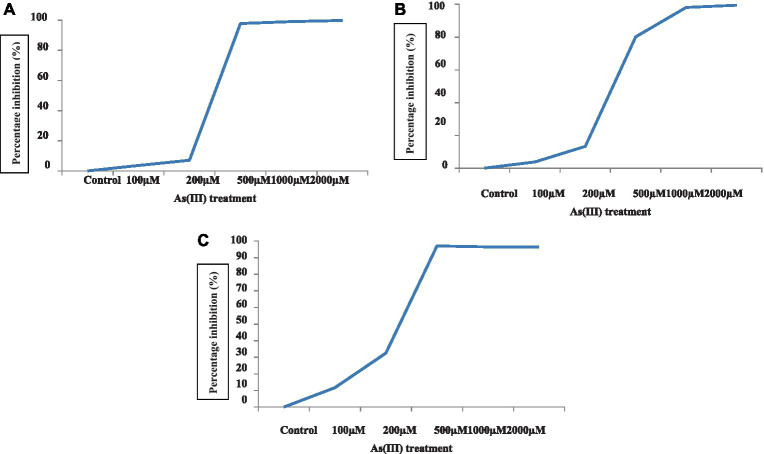
Percent inhibition of algae *C. vulgaris* **(A)**, *Nannochloropsis* sp. **(B)**, and *Anabaena doliolum* **(C)** treated with different concentrations of As(III).

The growth of blue-green algae at different concentrations of As(III) showed a gradual increase, followed by a slow decrease in growth in terms of the decreasing optical density at a 700 nm wavelength that resulted in a sigmoid growth response curve to some extent, which was obtained in all cases of growth at different durations. The growth response curves of the algae *C. vulgaris, Nannochloropsis* sp., and *A. doliolum* have been depicted in Figure 1. The alga *C. vulgaris* showed diminished growth at the higher concentration of As(III), i.e., 500, 1,000, and 2,000 μM. The survival of algae was observed up to the concentration of 200 μM. The exponential phase of the algae lasted for 6 days, whereas the lag and log phase spans only lasted 2 days. After the 12 days, saturation in growth of the algae was achieved. A similar pattern of growth was also observed in the case of *Nannochloropsis* sp. and *A. doliolum;* however, after 10 days of the experiment, growth was recovered in the *Nannochloropsis* sp. culture treated with 500 μM of As(III). The growth inhibition of algae (%) under different concentrations of As(III) is depicted in Figure 2. The results showed that with the increased concentration of As(III), a significant decrease in growth was observed.

### 3.2. Average and specific growth rate

The growth rate in the form of average and specific growth rate was calculated to observe the growth responses of algae under different As(III) treatments (Table 1). The specific growth rate of *C. vulgaris* was negative at 500, 1,000, and 2,000 μM of As(III). In the case of *Nannochloropsis* sp. and *A. doliolum*, a similar trend was observed. The maximum average growth rates in all the studied algae were observed at 200 μM of As(III). However, the average growth rate was found to be positive at 500 μM of As(III) in *Nannochloropsis* sp. (0.619) and *A. doliolum* (0.02) at 1,000 μM of As(III).

**Table 1:** Average growth rate (d^−1^) and specific growth rate (h^−1^) of the algae *C. vulgaris*, *Nannochloropsis* sp., and *A. doliolum* treated with different concentrations of As(III).

Name of the Algae		As(III) concentration (μM)					
	Growth analysis	Control	100	200	500	1,000	2,000
*C. vulgaris*	Average growth rate	1.85	1.87	1.88	−0.14	−0.20	−0.16
	Specific growth rate	0.79	0.80	0.74	0	−0.03	−0.022
*Nannochloropsis* sp.	Average growth rate	1.42	1.47	1.93	0.61	−0.48	−0.23
	Specific growth rate	0.56	0.57	0.85	−0.16	−0.14	−0.02
*A. doliolum*	Average growth rate	1.14	1.20	1.40	−0.15	0.020	0.16
	Specific growth rate	0.29	0.29	0.32	−0.34	−0.15	−0.10

### 3.3. Arsenic volatilization

**Figure 3:**
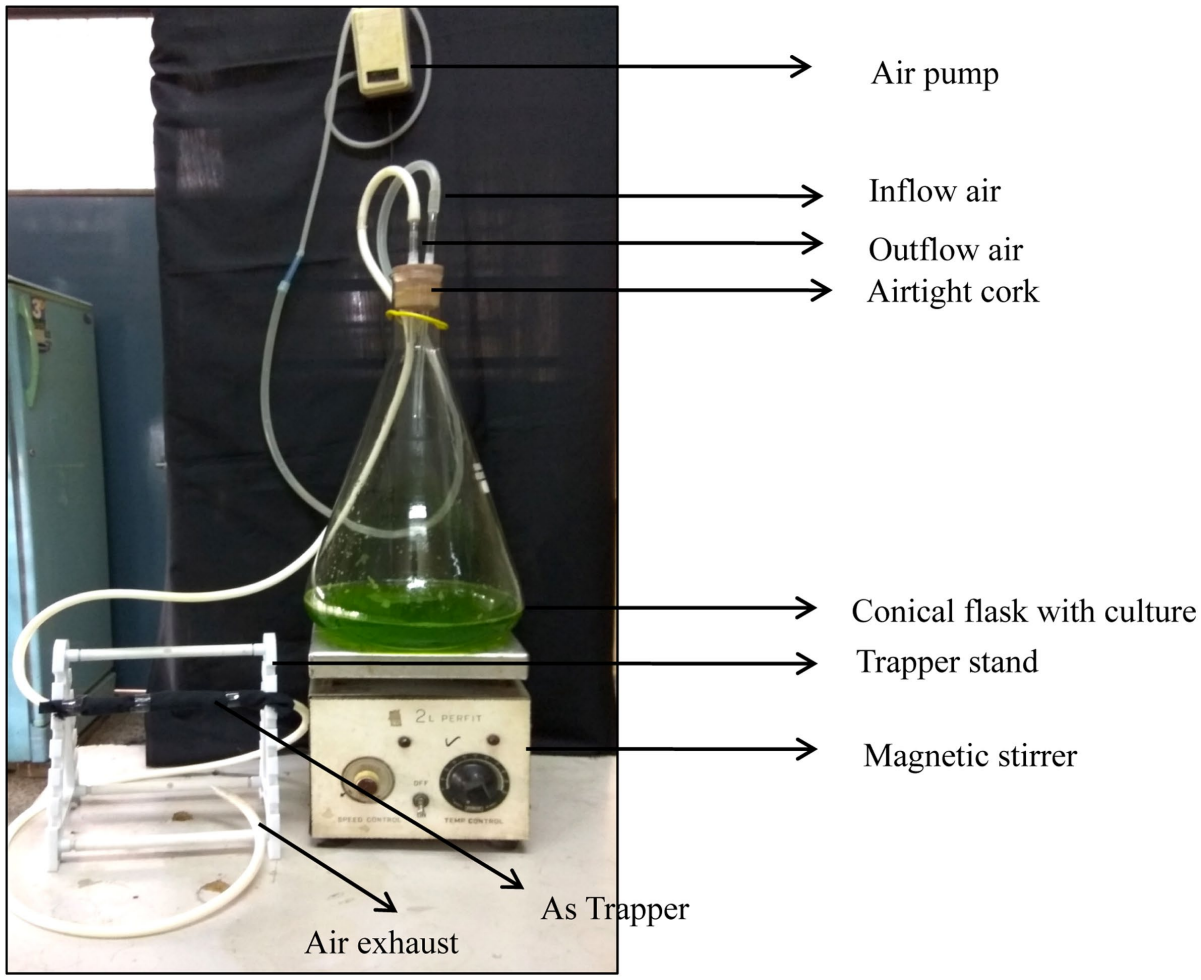
Cost-effective laboratory-designed model representation for determination of As volatilization.

The ability of the algae to produce volatile As in a pure culture was examined by applying various concentration of As(III) in a laboratory-designed model (Figure 3); it can be deduced that the Lc50 of all the algae was 200 μM. The growth and toxicity results demonstrated that selected algae can grow and tolerate up to 200 μM As(III).

A volatile As compound was detected in all the cultures grown in As-supplemented media at different durations of time (Table 2). The volatilization of the microalga *C. vulgaris* represented a duration-dependent increase in volatilized products. *C. vulgaris* treated with 200 μM of As(III) volatilized 1524.6 ng As at 7d of the treatment, which was increased to 2479.2 and 4382.75 ng at 14 and 21 days, respectively. In the case of *Nannochloropsis* sp., it was 1,488, 2,581.8g, and 4,393 ng at 7, 14, and 21 days, respectively. The blue-green alga *A. doliolum* volatilized 1224.3, 1732.5, and 2687.21 ng, respectively. The results comprehensively explain that the alga *Nannochloropsis* sp. significantly volatilized As at 21 days of the experiment.

**Table 2:** Volatilized As (ng) in different algae treated with 200 μM As(III) in different durations of time.

S. no.	Sample name	Duration (days)		
		7 days	14 days	21 days
1	*C. vulgaris*	1524.62 ± 16.22	2479.19 ± 16.48	4382.75 ± 10.25
2	*Nannochloropsis* sp.	1,488 ± 9.80	2581.79 ± 23.10	4392.99 ± 7.92
3	*A. doliolum*	1224.31 ± 6.60	1731.48 ± 11.18	2687.21 ± 7.93

All the values are means ± SD.

### 3.4. Effect on glutathione

**Figure 4:**
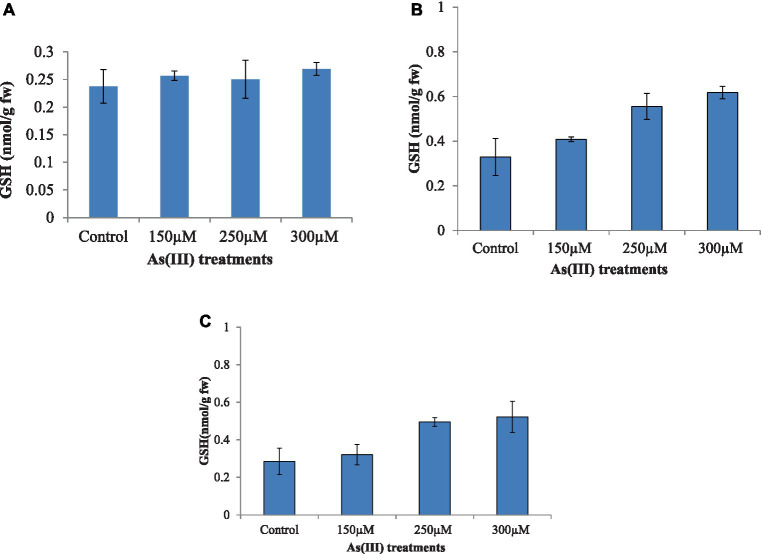
Effect of As(III) content in the antioxidant GSH in *C. vulgaris* **(A)**, *Nannochloropsis* sp. **(B)**, and *Anabaena doliolum* **(C)** treated with different concentrations of As(III). All values are means ± SD.

Algae treated with different concentrations of As(III) showed a marked increase in GSH level with the increased As(III) concentration (Figure 4). Maximum GSH concentration was observed with the alga *Nannochloropsis* sp., followed by *A. doliolum* and *C. vulgaris*, at 300 μM of As(III) in comparison to the control. The GSH level increased by 5.48, 69.2, and 73.6% in the case *C. vulgaris*, *Nannochloropsis* sp., and *A. doliolum*, respectively, at 250 μM of As(III) as compared to their respective controls. However, a slight decrease in GSH content (0.250 nm/g fw) was observed in *C. vulgaris* at 250 μM of As(III) in comparison to 150 μM of As(III) (0.256 nm/g fw).

### 3.5. Effect on pigment content

In the present study, the chlorophyll content of microalgae *Nannochloropsis*, *Chlorella vulgaris,* and *Anabaena doliolum* was analyzed, and this is depicted in Table 3. The results showed that a concentration-wise decreasing trend of chlorophyll was observed in all the studied microalgae under As stress. A maximum decrease in total chlorophyll content was observed in the microalgae treated with 300 μM As(III) in the order of 42.25% (*Nannochloropsis* sp.), 45.55% (*Chlorella vulgaris*), and 28.57% (*Anabaena doliolum*) in comparison to the control.

**Table 3:** Effect on pigment content (chlorophyll a, chlorophyll b, and total chlorophyll) in microalgae *Nannochloropsis* sp., *Chlorella vulgaris*, and *Anabaena doliolum* treated with different As(III) concentrations.

S.no.	Treatment (AsIII)	*Nannochloropsis* sp.			*Chlorella vulgaris*			*Anabaena doliolum*		
		Chl a	Chl b	Total Chl	Chl a	Chl b	Total Chl	Chl a	Chl b	Total Chl
1	Control	0.0050 ± 0.0009	0.0020 ± 0.0006	0.0071 ± 0.0007	0.0063 ± 0.001	0.0048 ± 0.0006	0.011 ± 0.001	0.008 ± 0.001	0.0057 ± 0.0003	0.014 ± 0.001
2	150 μM	0.0047 ± 0.0006	0.0021 ± 0.0002	0.0069 ± 0.0006	0.0056 ± 0.0008	0.0026 ± 0.0001	0.0083 ± 0.0008	0.005 ± 0.001	0.0048 ± 0.0006	0.010 ± 0.001
3	250 μM	0.0032 ± 0.0008	0.0020 ± 0.0003	0.0052 ± 0.0007	0.0049 ± 0.0004	0.0030 ± 0.0004	0.0079 ± 0.0004	0.004 ± 0.001	0.0044 ± 0.0001	0.009 ± 0.0002
4	300 μM	0.0033 ± 0.0001	0.0011 ± 0.0006	0.0044 ± 0.0001	0.0038 ± 0.0005	0.0021 ± 0.0003	0.0060 ± 0.0005	0.005 ± 0.0001	0.0047 ± 0.0008	0.010 ± 0.0001

All the values (mg/g fw) are means ± SD (*n* = 3).

## 4. Discussion

### 4.1. Growth and toxicity responses of algae

Microbes are diverse habitat organisms endowed with untapped potential of biotransformation, accumulation, oxidation, and reduction (Meng et al., 2011) to diminish the toxic effect of organic and inorganic contaminants present in the environment. As is one of the potent carcinogens worldwide, and its natural attenuation through a photosynthetic microorganism may contribute to As remediation and As biogeochemical cycling (Yin et al., 2011a,b). Algae treated with different concentrations of As(III) show reduced growth. The growth and biomass of algae are intimately correlated with the phenomenon of photosynthesis and production of energy (Qian et al., 2018) and thus act as a marker of stress and an important way of expressing the ecological success of a species or strain in adapting to its natural environment (Rai et al., 2013). The decreased growth rate in the different algae in the present study may be attributed to chlorosis and chloroplast degeneration due to increased photoreduction leading to the formation of triplet Chl (Ball et al., 2004), which reacts with ^3^O_2_ and forms ^1^O_2_ and has a strong damaging effect on PS I, PS II, and photosynthetic machinery (Gill and Tuteja, 2010). A decreased growth rate in *Nannochloropsis* sp. under As(III) has also been reported by Upadhyay et al. (2016).

### 4.2. Volatilization of As in three photosynthetic microorganisms

Microbial volatilization is the complex cascade of biotransformation, methylation, and redox reactions for the conversion of toxic inorganic contaminants into less toxic organic and volatile compounds (Rahman and Hassler, 2014). As volatilization includes the oxidation of As(III) to As(V) followed by the production of organo-arsenicals and volatile As species {trimethylarsine [TMAs(III)] gas} (Bentley and Chasteen, 2002; Cullen et al., 2016). In the present study, microalgae *Nannochloropsis* sp. showed maximum volatilization potential as compared to *C. vulgaris* and *A. doliolum*. High volatilization capacity distinctly unveils the high adaptation and tolerance potential against a harsh environment (Suhendrayatna et al., 1999). The occurrence of volatilized As might be the result of well-established metabolic activities of the algae and other microorganisms, as reported by Pearce et al. (1998) in fungi. Hussain et al. (2021) also reported that microorganisms have the potential to solubilize, transform, and absorb/adsorb toxic elements, making them suitable for the remediation of toxic elements present in the environment. They also reported the biotransformation potential of microalgae.

### 4.3. Effect on antioxidant glutathione

Glutathione, a thiol buffer present in cytosol, modulates environmental stress via glutathionylation (Zaffagnini et al., 2012), a posttranslational response against oxidative damage. The As(III)-coupled tolerance response by GSH might be ascribed to GSH being the only known SH-containing antioxidant compound, which responds significantly against As(III) toxicity by binding with the sulfhydryl group, the formation of an As(GS)_3_ complex, and extrusion via an ABC transporter outside the cell (Scott et al., 1993). In the present study, the study of glutathione study considered due to its potential role in As(III) metabolism and its presence in all the cells at higher concentrations. The increased GSH level in the present study reduced the *in vivo* level of As(III), thus protecting the algae from oxidative injury (Thomas, 2008). Moreover, the results suggest that As methylation is an As(III)-dependent process that contributes as a substrate for methylating enzymes. It has been reported that in microalgae, GSH forms a complex compound with As(III) (arsenotriglatathione), which leads to detoxifying the toxicity through the process of the biotransformation of As into arsenosugar, arsenolipid, and methylated As species. Further, studies have reported that GSH is requisite for the methylation of As, and the process of As(III) methylation occurs non-enzymatically in the presence of GSH (Hayakawa et al., 2005). Thus, the higher the GSH in the aquatic environment, the higher the chance of As(III) methylation and detoxification in microalgae (Yin et al., 2011a,b).

### 4.4. Implication of the arsenic-treated microalgae with reference to growth, pigment synthesis, and production

Microalgae growth is linked to the synthesis of photosynthetic pigments. Good growth of the microalgae indicates better photosynthesis, pigment content, and the production of starch necessary for microalgal intensification. In the present study, better growth of microalgae under As stress demonstrated their health status. Chlorophyll is a high-value biological compound, and various research has focused on enhancing the synthesis of pigments by the application of various environmental stresses, culture conditions, and nutrient alterations. Microalgae grown under an As environment showed a sort of toxicity; however, a good specific growth rate and absorption peak (measured by determining the density of the microalgal cell corresponding to the chlorophyll pigment) reflect good pigment content in microalgae. Thus, the microalgae *Nannochloropsis*, *Chlorella vulgaris,* and *Anabaena doliolum* could be employed as organisms with dual benefits, as an arsenic remediator and pigment feedstock; however, ecotoxicological analysis is a prerequisite to their industrial and large-scale application. The decreased chlorophyll content in the microalgae indicated the toxicity exerted by As, which may be ascribed to chlorosis, membrane damage, photosystem II bleaching, and impairment of photosynthetic machinery (Upadhyay et al., 2016, 2021). Similar results of decreased pigment content under various environmental stresses in *microalgae* were also reported by various other authors (Elleuch et al., 2021).

## 5. Conclusion

The present study revealed the phytovolatilization capability of the algae *C. vulgaris, Nannochloropsis* sp., and *A. doliolum* grown in an As-contaminated environment, finding that they could tolerate up to 200 μM As(III). The model designed for the volatilization study proved to be a sustainable and cost-effective way to detect volatile As derivatives in a laboratory. Thus, the algae may be employed as environmentally friendly entities for the phytoremediation, biotransformation to less toxic forms, and volatilization of As and other metals/metalloids, which might contribute to decontaminating the environment.

## Data availability statement

The original contributions presented in the study are included in the article/supplementary material, further inquiries can be directed to the corresponding author.

## Author contributions

AU, SM, RS, LS, NS, RPS, and AS wrote the manuscript under the mentorship of GS. All the authors read the manuscript for the final submission.

## Conflict of interest

The authors declare that the research was conducted in the absence of any commercial or financial relationships that could be construed as a potential conflict of interest.

## Publisher’s note

All claims expressed in this article are solely those of the authors and do not necessarily represent those of their affiliated organizations, or those of the publisher, the editors and the reviewers. Any product that may be evaluated in this article, or claim that may be made by its manufacturer, is not guaranteed or endorsed by the publisher.
